# Viral RNase3 Co-Localizes and Interacts with the Antiviral Defense Protein SGS3 in Plant Cells

**DOI:** 10.1371/journal.pone.0159080

**Published:** 2016-07-08

**Authors:** Isabel Weinheimer, Tuuli Haikonen, Marjo Ala-Poikela, Mirko Moser, Janne Streng, Minna-Liisa Rajamäki, Jari P. T. Valkonen

**Affiliations:** 1 Department of Agricultural Sciences, University of Helsinki, Helsinki, Finland; 2 Foundation Edmund Mach, San Michele, Italy; Louisiana State University, UNITED STATES

## Abstract

*Sweet potato chlorotic stunt virus* (SPCSV; family Closteroviridae) encodes a Class 1 RNase III endoribonuclease (RNase3) that suppresses post-transcriptional RNA interference (RNAi) and eliminates antiviral defense in sweetpotato plants (*Ipomoea batatas*). For RNAi suppression, RNase3 cleaves double-stranded small interfering RNAs (ds-siRNA) and long dsRNA to fragments that are too short to be utilized in RNAi. However, RNase3 can suppress only RNAi induced by sense RNA. Sense-mediated RNAi involves host suppressor of gene silencing 3 (SGS3) and RNA–dependent RNA polymerase 6 (RDR6). In this study, subcellular localization and host interactions of RNase3 were studied in plant cells. RNase3 was found to interact with SGS3 of sweetpotato and *Arabidopsis thaliana* when expressed in leaves, and it localized to SGS3/RDR6 bodies in the cytoplasm of leaf cells and protoplasts. RNase3 was also detected in the nucleus. Co-expression of RNase3 and SGS3 in leaf tissue enhanced the suppression of RNAi, as compared with expression of RNase3 alone. These results suggest additional mechanisms needed for efficient RNase3-mediated suppression of RNAi and provide new information about the subcellular context and phase of the RNAi pathway in which RNase3 realizes RNAi suppression.

## Introduction

*Sweet potato chlorotic stunt virus* (SPCSV, genus *Crinivirus*; Closteroviridae) predisposes sweetpotato (*Ipomoea batatas*) to heavy yield losses by eliminating the basal antiviral defense based on post-transcriptional RNA interference (RNAi), which also renders plants susceptible to other unrelated viruses [1]. The dsRNA-specific Class 1 RNase III endoribonuclease (RNase3) encoded by SPCSV is sufficient for eliminating antiviral defense when expressed from a transgene in sweetpotato plants [2]. RNase3 can suppress sense RNA–mediated RNAi (gene co-suppression) but not RNAi induced by antisense RNA or dsRNA [2–4]. RNase3 can process long dsRNA and double-stranded short interfering RNAs (siRNA) in a length- and sequence-independent manner [2, 3], but certain structural anti-processing determinants (asymmetric bulges) typical for microRNA (miRNA) duplexes may interfere with processing [5]. Taken together, RNase3 can potentially target the dsRNA substrates of the Dicer-like dsRNA-specific endoribonucleases (DCL1-4), as well as the 21-, 22-, and 24-nucleotide (nt) siRNA duplexes produced by DCLs in plants and which guide the RNA-induced silencing complex (RISC) to target and cleave homologous RNAs [6].

RNA-dependent RNA polymerases (RDRs) are important in sense-mediated RNAi. They can copy ssRNA to dsRNA, which is subsequently processed to siRNA by DCLs. In this process, secondary siRNAs are produced also from regions outside the primary targeted area, which is called transitivity and enhances antiviral RNAi [7, 8]. Previous studies found no influence of RNase3 on transitivity [3]. SGS3 is another plant protein involved in antiviral defense, but the mechanism is not well understood. For example, a lowered level of SGS3 mRNA enhances susceptibility of *Arabidopsis thaliana* (L.) Heynh. to *Cucumber mosaic virus* (genus *Cucumovirus*) but not *Turnip vein clearing virus* (genus *Tobamovirus*) or *Turnip mosaic virus* (genus *Potyvirus*) [9, 10]. In potyvirus infection, SGS3-silencing reduces accumulation of viral RNA, as reported with *Potato virus A* and *Soybean mosaic virus* strain G7 [11, 12].

The coordinated functions of SGS3 with RDR6 are pivotal in trans-acting siRNA (tasiRNA) pathways that regulate plant gene expression. The first step in the tasiRNA pathway is the miRNA-programmed cleavage of tasiRNA gene (*TAS*) transcripts, which are stabilized by SGS3 [13]. For example, *TAS3* is conserved among plant species [14] and has two miRNA390 target sites, of which the 3’ site is recognized and cleaved specifically by RISC containing the RNase H–like endoribonuclease Argonaute 7 (AGO7). Subsequently, RDR6 converts the 5’-cleavage fragment of *TAS3* transcripts to dsRNA in SGS3/RDR6 bodies (also called siRNA bodies), and DCL4 processes the dsRNA to 21-nt siRNAs. Consistent with these functions, AGO7 co-localizes with the SGS3/RDR6 bodies in the cytoplasm [15, 16].

Involvement of RDR6 and SGS3 in RNAi suggests that plant viruses may have evolved mechanisms to interfere with their functions. Indeed, the protein P6 of *Rice yellow stunt virus* (genus *Nucleorhabdovirus*) interacts with RDR6 of rice (*Oryza sativa*) and interferes with systemic silencing of RNAi, but cannot suppress RNAi locally in the leaves in which silencing is induced [17]. Therefore, it differs from RNase3 that suppresses RNAi locally [2]. On the other hand, the triple gene block protein (TGBp1) of *Plantago asiatica mosaic virus* (genus *Potexvirus*) interacts with both RDR6 and SGS3 to mediate their aggregation and inhibits SGS3/RDR6-dependent dsRNA synthesis and tasiRNA accumulation [18]. Similarly, the P protein of *Lettuce necrotic yellows virus* (genus *Cytorhabdovirus*) interacts with both RDR6 and SGS3 in large protein aggregates, which inhibits RNAi amplification [19]. Furthermore, the RNAi-suppressing proteins V2 of *Tomato yellow leaf curl virus* (ssDNA genome, genus *Begomovirus*) [20], p2 of *Rice stripe virus* (negative-sense ssRNA genome; genus *Tenuivirus*) [21] and VPg of *Potato virus A* (ssRNA genome, genus *Potyvirus*) [11] interact with the SGS3 proteins of tomato (*Solanum lycopersicum* L.), rice (*Oryza sativa* L.), and potato (*Solanum tuberosum* L.), respectively.

RNase3 is a unique suppressor interfering with RNAi in an endoribonuclease activity-dependent manner. However, little is known about the subcellular localization and host interactions of RNase3 in plant cells. RNase3 interferes with sense-mediated RNAi but is unable to suppress RNAi induced by hairpin RNA [2], similar to the TGBp1, P, V2, p2 and VPg proteins mentioned above [11, 18–21]. Therefore, the aim of this study was to examine possible interactions of RNase3 with SGS3 and RDR6 and interference with the RNAi pathway involving these host proteins.

## Results

### Subcellular localization of RNase3 in cytoplasm and nucleus

**Fig 1:**
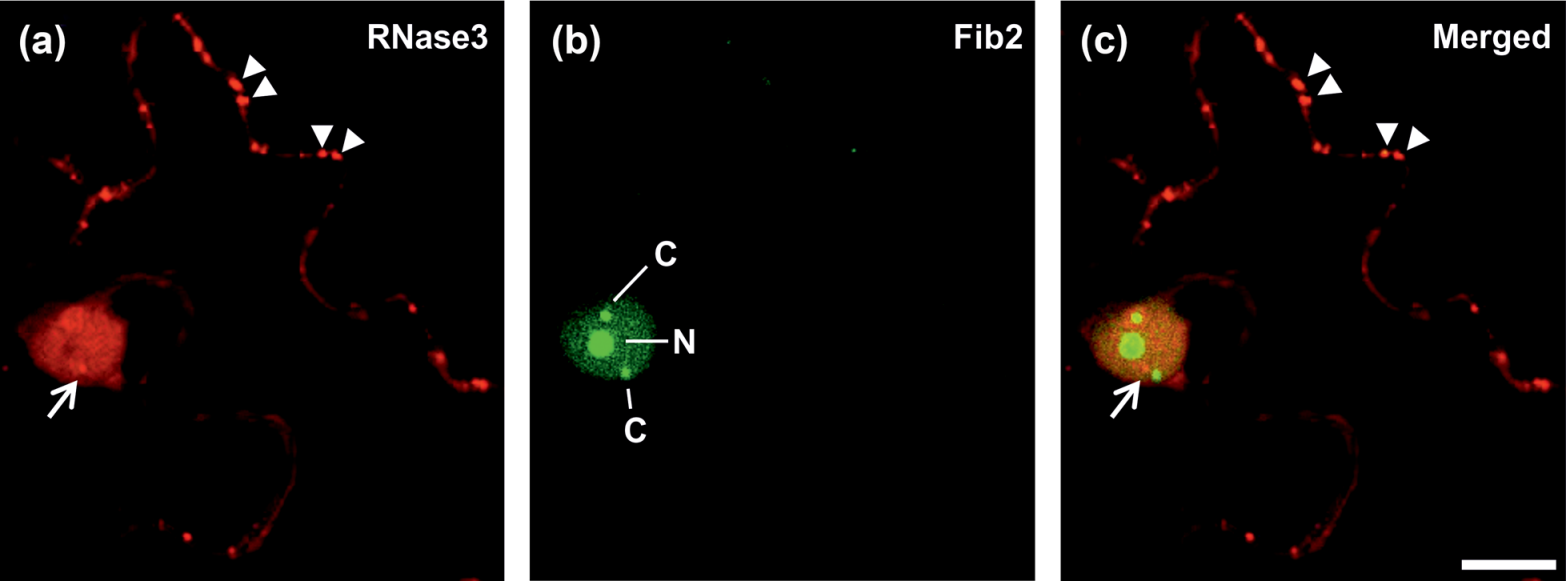
Subcellular localization of RNase3 in epidermal cells of *Nicotiana benthamiana* following expression by agroinfiltration. (a) RNase3-dsRED (red signals) was detected in cytoplasmic punctate bodies by confocal microscopy at 2 dpi. A few of the many bodies are pointed out with arrowheads. It was also present in the nucleus and subnuclear bodies (arrow). (b) Fibrillarin of *A*. *thaliana* (Fib2) was expressed as a fusion with GFP (green) and used as a marker for nucleolus (N) and Cajal bodies (C). (c) Merged image revealed localization of RNase3 in subnuclear bodies (arrow) other than nucleolus or Cajal bodies. Scale bars, 10 μm.

Agroinfiltration was used to co-express RNase3 (RNase3-dsRED; red fluorescent protein fused to the C-terminus) in leaves of *Nicotiana benthamiana* Domin along with fibrillarin (Fib2-GFP, green fluorescent protein fused to the C-terminus), which is a major nucleolar protein also present in Cajal bodies [22, 23]. Confocal microscopy of epidermal cells in the infiltrated areas at 2 days post-infiltration (dpi) revealed dsRED signals in punctate bodies in the cytoplasm (arrowheads, Fig 1A and 1C) and in the nucleus, and also in subnuclear bodies that differed from Cajal bodies (arrow in Fig 1A and 1C). The nucleolus and Cajal bodies did not show pronounced dsRED signals.

### Co-localization of RNase3 and RDR6

**Fig 2:**
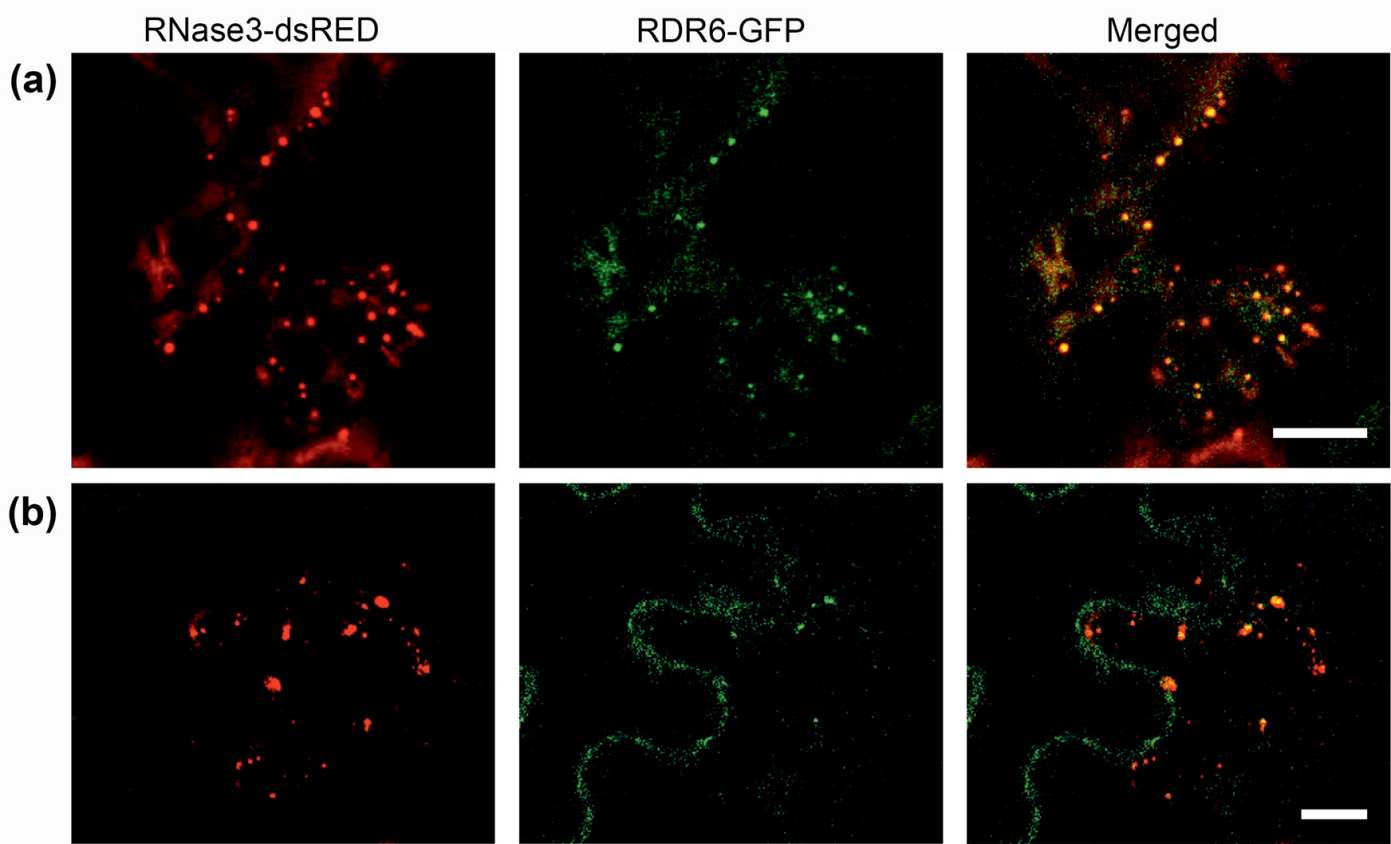
Co-localization of RNase3 and RDR6 in epidermal cells of *N*. *benthamiana* following co-expression by agroinfiltration. The red signals of RNase3-dsRED and green signals of (a) AtRDR6-GFP (*A*. *thaliana*) and (b) NtRDR6-GFP (*N*. *tabacum*) co-localized in cytoplasmic, punctate bodies detected by confocal microscopy at 2 dpi. Images in (a) and (b) illustrate optical planes in which many RDR6-containing bodies were observed. Scale bars, 10 μm.

The punctate RNase3-containing bodies detected in *N*. *benthamiana* epidermal cells resembled the SGS3/RDR6 bodies detected in *A*. *thaliana* and *Nicotiana tabacum* L. [15, 16]. Therefore, RNase3-dsRED was co-expressed with the RDR6 of *A*. *thaliana* or *N*. *tabacum*, both with GFP fused to the C-terminus (AtRDR6-GFP and NtRDR6-GFP, respectively), in leaves of *N*. *benthamiana* by agroinfiltration. Confocal microscopy showed that signals of AtRDR6-GFP (Fig 2A) and NtRDR6-GFP (Fig 2B) were observed in punctate cytoplasmic bodies that co-localized with the RNase3-dsRED-positive punctate bodies. Co-localization of AtRDR6 and NtRDR6 with RNase3 was also observed in protoplasts prepared from the agroinfiltrated leaf tissues (S1 Fig).

Substitution of Asn37 and Glu44 with alanine in the catalytic site of RNase3 results in an RNase3-Ala mutant lacking dsRNA cleavage activity [3]. Localization studies carried out with RNase3-Ala fused with dsRED revealed signals in similar punctate bodies and similar co-localization with RDR6 (S2 Fig) as found with RNase3 (Fig 2).

### The RNase3-SGS3 complex co-localizes with SGS3/RDR6 bodies

**Fig 3:**
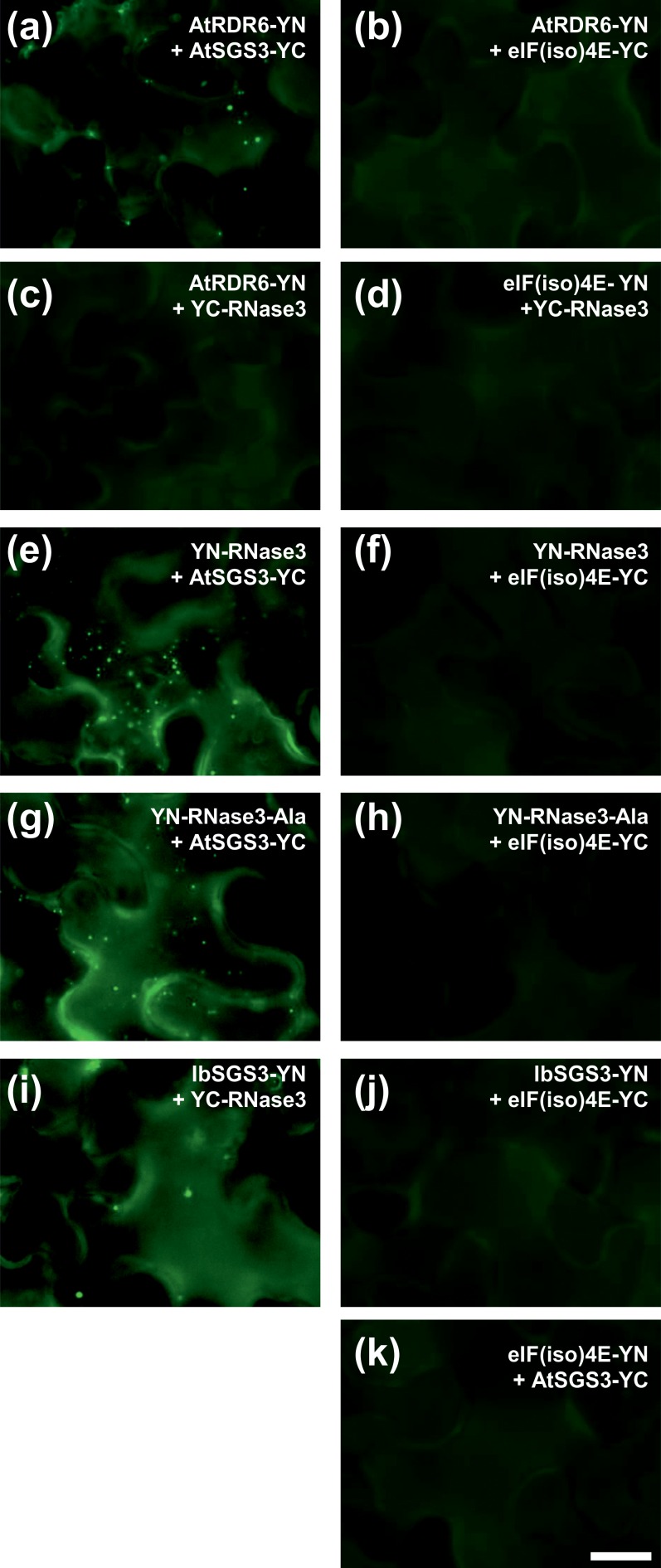
Interactions of SGS3 with RDR6 and RNase3 *in planta*. Protein interactions were tested by BiFC and monitored in epidermal cells of *N*. *benthamiana* using epifluorescence microscopy. The name of the protein indicates whether the YFP half was fused to the N- or C-terminus of the test protein (e.g., YN-RNase3 and IbSGS3-YC, respectively). Co-expression of the test proteins with eIF(iso)4E of potato [27] was included as a negative control for interaction (panels b, d, f, h, j and k). (a) Interaction of AtRDR6 and AtSGS3 resulted in SGS3/RDR6 bodies (positive control); (b) negative control. (c) RNase3 did not interact with AtRDR6, but (e) interacted with AtSGS3 (*A*. *thaliana*) and (i) IbSGS3 (*I*. *batatas*). (g) Catalytically inactive RNase3-Ala was also able to interact with SGS3. Data were collected at 2 dpi. All images were acquired at the same magnification. Scale bar, 20 μm.

Co-localization of RNase3 and RDR6 prompted us to test the possible interactions of RNase3 with RDR6 or SGS3, which was done using bimolecular fluorescence complementation (BiFC) [24, 25]. *AtRDR6* and *AtSGS3* of *A*. *thaliana* and *RNase3* were introduced to BiFC vectors and expressed in translational fusion with the N- or C-terminal halves of yellow fluorescent protein (YFP; YN or YC, respectively). BiFC assay of these proteins with eIF(iso)4E served as a negative control (Fig 3, right panel). Co-expression of AtRDR6 with AtSGS3 served as a positive interaction control in the experiments and resulted in yellow fluorescence confined to cytoplasmic punctate bodies (Fig 3A), as expected [15].

RNase3 did not interact with AtRDR6 (Fig 3C). In contrast, interaction of RNase3 with AtSGS3 was readily observed in cytoplasmic punctate bodies (Fig 3E). Similarly, RNase3-Ala interacted with AtSGS3 (Fig 3G). The SGS3 coding sequence of sweetpotato (cv. Huachano) (designated IbSGS3) was cloned utilizing the transcriptome data of sweetpotato cv. Xushu18 available in NCBI Sequence Read Archive (accession number SRX090758; [26]) (see Methods and S3 Fig) and introduced into the BiFC vectors. IbSGS3 interacted with RNase3 in punctate bodies (Fig 3I) similar to those observed with the AtSGS3-RNase3 interaction (Fig 3E).

**Fig 4:**
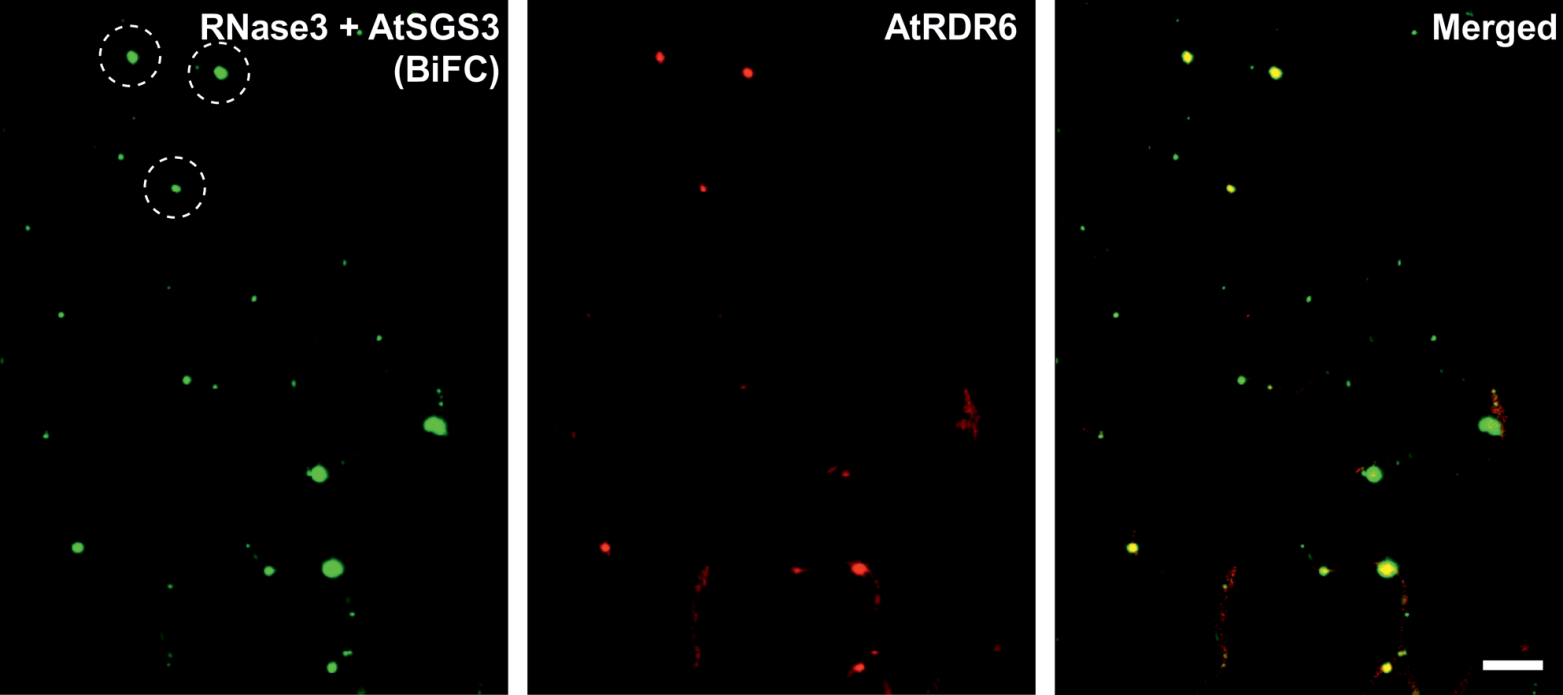
Interactions of RNase3 and AtSGS3, as assessed with BiFC and confocal microscopy, co-localize with cytoplasmic punctate bodies containing AtRDR6 in epidermal cells of *N*. *benthamiana*. The three proteins were co-expressed by agroinfiltration. Images were acquired at an optical plane in which many RDR6-containing bodies were observed at 2 dpi. The dashed circles point out some of the cytoplasmic punctate bodies that show signals of the RNase3-SGS3 interaction and co-localize with RDR6. Scale bar, 10 μm.

Co-expression of YN-RNase3, AtSGS3-YC, and AtRDR6-mRFP revealed that cytoplasmic punctate bodies expressing BiFC signals (green) of the RNase3-SGS3 interaction co-localized with the signals (red) of RDR6-containing bodies (Fig 4). Taken together, the results indicated that RNase3 interacts with SGS3 and hence co-localizes with SGS3/RDR6 bodies. The catalytic site mutations in RNase3-Ala did not affect these interactions and subcellular localization.

### Co-expression of RNase3 and IbSGS3 enhances suppression of RNAi

**Fig 5:**
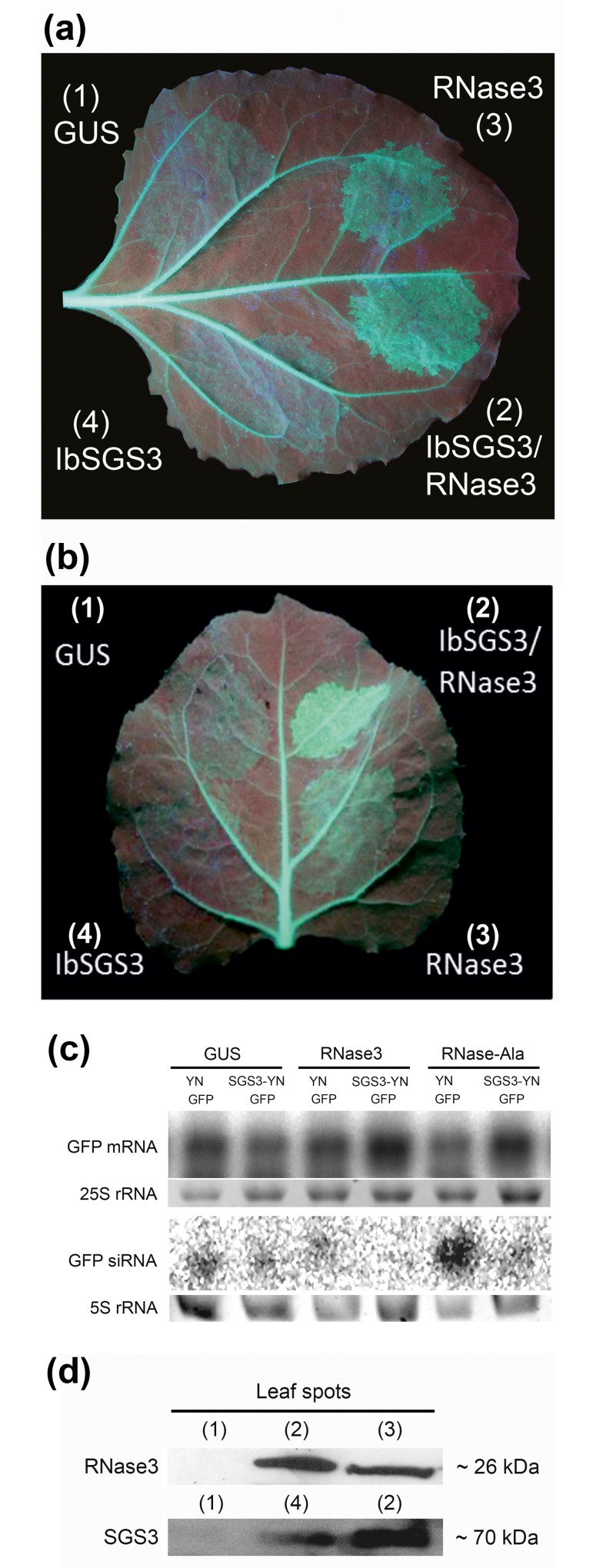
Co-expression of RNase3 and IbSGS3 enhances suppression of sense-mediated gene silencing (gene co-suppression). (a) and (b) Four sectors (1 to 4) of a leaf of *N*. *benthamiana* 16c constitutively expressing *gfp* were agroinfiltrated to co-express GFP and (1) GUS (negative control), (2) IbSGS3 and RNase3, (3) RNase3, or (4) IbSGS3. If RNase3 was not used, the corresponding *Agrobacterium* strain was replaced with a strain expressing β-glucuronidase (GUS, negative control). IbSGS3 was expressed with the N-proximal part of YFP fused to the C-terminus. If it was not used, it was replaced with an *Agrobacterium* strain expressing YN (N-proximal half of *yfp*) to maintain similar sense-mediated silencing pressure. The treatments are positioned differently in the two leaves in terms of the younger (basal) and older (tip) part of the leaf. Silencing of *gfp* was observed by the disappearance of GFP fluorescence (sectors 1 and 4), whereas GFP fluorescence above the background level indicated suppression of *gfp* silencing (sectors 2 and 3). The leaf was photographed under UV light at 6 dpi. Similar results were obtained in five independent experiments. (c) Northern analysis of *gfp* mRNA and *gfp* mRNA-derived siRNA in the agroinfiltrated leaf tissues. Co-expression of GUS or IbSGS3 with GFP by agroinfiltration in *gfp*-transgenic leaves resulted in *gfp* silencing, as shown by the readily detectable accumulation of *gfp*-derived siRNA (Fig 5C). In contrast, co-expression of GFP and RNase3 resulted only in low accumulation of *gfp* siRNA, and no *gfp* siRNA could be detected following co-expression of GFP, RNase3 and SGS3; however, accumulation of *gfp* mRNA was enhanced (Fig 5C). Co-expression of the RNase3-Ala mutant (disabled from catalytic activity on dsRNA) with GFP resulted in readily detectable accumulation of *gfp* siRNA, whereas co-expression of RNase3-Ala, SGS3 and GFP resulted in low accumulation of *gfp* siRNA (Fig 5C). 25S and 5S ribosomal RNA is shown as a loading control, respectively. (d) Western analysis of RNase3 and IbSGS3-YN in the agroinfiltrated leaf sectors illustrated in (a) by immunoblotting using anti-RNase3 and anti-GFP antibodies, respectively. Molecular masses of the detected proteins (kDa) were estimated by comparison with the protein marker run in the gel.

RNase3 can suppress sense-mediated RNAi, *e*.*g*., when silencing of the constitutively expressed *gfp* transgene is induced in *N*. *benthamiana* 16c following *gfp* overexpression by agroinfiltration [2]. Influence of IbSGS3, RNase3 and RNase3-Ala (a mutant of RNase3 debilitated for catalytic activity on dsRNA), as well as the effect of IbSGS3 co-expressed with RNase3 or RNase3-Ala, on sense-mediated *gfp* silencing was tested in *N*. *benthamiana* 16c. If RNase3 or RNase3-Ala was not used, the corresponding *Agrobacterium* strain was replaced with a strain expressing β-glucuronidase (GUS, negative control). Because the YN-tagged IbSGS3 (SGS3-YN) was used in these experiments, it was replaced, when not used, by an *Agrobacterium* strain expressing YN, so to maintain the sense-mediated *gfp* silencing pressure similar in all treatments. Following co-expression of *gfp*, *YN* and *GUS*, GFP fluorescence initially increased, but then decreased substantially by 6 dpi (Fig 5A and 5B), indicating no significant suppression of silencing. Similar results were obtained following co-expression of *gfp* and *IbSGS3*. These results were consistent with detectable accumulation of *gfp*-derived siRNA, as tested by northern analysis (Fig 5C). However, when leaf tissues were co-infiltrated with *Agrobacterium* strains for expression of GFP and RNase3, GFP fluorescence remained higher than the background fluorescence resulting from expression of the constitutively expressed *gfp* transgene of the 16c line (note the green fluorescent veins in Fig 5A and 5B) and the additional expression of the infiltrated *gfp*-expressing construct. GFP fluorescence was further enhanced in leaf tissue co-expressing GFP, IbSGS3, and RNase3 (Fig 5A and 5B). *gfp* mRNA levels were not much increased in leaf tissues co-infiltrated with *gfp* and *RNase3*, but they were clearly elevated in leaves co-infiltrated with *gfp*, *IbSGS3* and *RNase3*, as compared with leaf tissues infiltrated with *gfp* and *GUS*, or *gfp*, *GUS* and *IbSGS3* (Fig 5C). Accumulation of *gfp*-specific siRNAs was negatively correlated with *gfp* mRNA accumulation (Fig 5C) and was high in leaf tissues co-expressing *gfp* and *RNase3-Ala* (Fig 5C). In contrast, little *gfp*–derived siRNA was observed in leaf tissue co-expressing *gfp*, *RNase3-Ala* and *IbSGS3* although suppression of silencing could not be observed visually in the leaves (Fig 5C).

RNase3 and IbSGS3 were readily expressed in the tissues, as detected by western analysis (Fig 5D). These results were reproduced in five independent experiments, indicating enhanced suppression of gene silencing in the presence of both IbSGS3 and RNase3.

### tasiRNA production is not affected by RNase3

RNase3 has not been found to interfere with transitivity [3], but possible interference of RNase3 with the tasiRNA pathway has not been tested. Therefore, *TAS3* transcript–derived siRNA were analyzed in transgenic sweetpotato plants (cv. Huachano) expressing RNase3 [2]. Computational analysis of the Sequence Read Archive database sequences of sweetpotato cv. Xushu18 (SRX090758) revealed that the *A*. *thaliana* TAS3 5’D7(+) transcript-specific tasiRNA probe [28] aligned well with a number of reads, which allowed partial reconstruction of the putative *IbTAS3* transcript sharing high sequence identity with known *TAS3* transcripts. The *A*. *thaliana* D7(+)-specific probe was used to detect tasiRNA in sweetpotato cv. Huachano and two RNase3-transgenic lines, but no differences in the tasiRNA amounts were detected (S4 Fig).

## Discussion

Our results show that RNase3 co-localizes and is associated with SGS3/RDR6 bodies implicated in plant gene regulation and antiviral RNAi [9, 29]. The association is likely mediated by the interaction of RNase3 with SGS3, as indicated by BiFC assay. Deprivation of SGS3 can reduce RNAi-based virus resistance and enhance accumulation of cucumoviruses in plants [9], whereas accumulation of potyviruses correlates positively with SGS3 accumulation [11, 12]. In this respect it is noteworthy that co-expression of SGS3 with RNase3 slightly (but reproducibly) enhanced suppression of sense RNA–induced RNAi by RNase3. Yoshikawa *et al*. [13] have shown that in the micro-RNA (miRNA) directed process of trans-acting small interfering RNA (tasiRNAs) production from *TAS2* gene transcript by RISC, the 3' cleavage fragment of the *TAS2* transcript is protected from degradation. In this process, SGS3 binds to AGO1-RISC via the dsRNA formed by interaction of the miRNA with the target RNA. The authors also concluded that the SGS3-dependent stabilization of the 3' fragment of TAS2 RNA is crucial to tasiRNA production [13]. However, the results of our study may suggest that interaction of RNase3 with SGS3 is part of the protective functions of SGS3, or assists RNase3 in targeting and cleaving ds-siRNA or secondary viral dsRNA synthesized by RDR6. The catalytic activity on dsRNA is pivotal for suppression of RNAi by RNase3 and results in short fragments of ~14 base pairs that are too short to be incorporated in RISC and are inactive in RNAi [2, 4]. It is noteworthy that RNase3 was found to interact with the SGS3 homologs of two unrelated plant species, *A*. *thaliana* and sweetpotato, of which the latter is the natural host of SPCSV. These findings imply that RNase3-SGS3 interactions could be a common and an important mechanism for SPCSV to control antiviral RNAi in its host plants.

SGS3 and RDR6 are involved in tasiRNA pathways, but mutations in SGS3 or RDR6 do not always yield a discernable phenotypic change in *A*. *thaliana* [29, 30]. Hence, also interference of RNase3 with the tasiRNA pathways would not necessarily cause any phenotype, whereas perturbation of miRNA homeostasis more often is associated with morphological changes [31]. The transgenic sweetpotato plants expressing RNase3 do not show any phenotype different from wild-type plants, except more pronounced purple pigmentation in leaves, similar to SPCSV-infected plants. Therefore, we tested the possible influence of RNase3 on *TAS3* transcript–based tasiRNA production, which represents the most conserved tasiRNA gene in plants [14], but results were negative. Other tasiRNA pathways could be elucidated in sweetpotato and influence of RNase3 on them tested in future studies.

The punctate bodies containing RNase3, SGS3 and RDR6 were cytoplasmic, which is consistent with previous studies reporting occurrence of the SGS3/RDR6 bodies in cytoplasm without any specific association with other cellular bodies or organelles [15]. In addition, RNase3 was detected in the nucleus. RDR6 is also found in the nucleus, in contrast to the SGS3/RDR6 bodies [32]. The size of RNase3-dsRED (ca. 51 kDa) could allow passive diffusion to the nucleus [25], but the nuclear RNase3-containing bodies, which were distinct from Cajal bodies, are intriguing and suggest that RNase3 might have a functional role in the nucleus. In general, recent studies question the view that post-transcriptional RNAi would occur only in the cytoplasm. dsRNA directed to an intron can silence a gene [32] and DCL4 responsible for production of the majority of the virus-derived siRNAs in *A*. *thaliana* [33] is detected only in the nucleus [15, 16, 32]. Furthermore, nuclear import of the P6 protein of *Cauliflower mosaic virus* is required for the suppression of antiviral RNAi [34]. Therefore, it is likely that antiviral RNAi occurs in both the cytoplasmic and nuclear compartments [32]. Finally, the studies of Garcia-Ruiz *et al*. [33] have shown that basal levels of antiviral RNAi and siRNA biogenesis remain in *Arabidopsis* mutants lacking RDR1, RDR2 and RDR6, which suggests that there is an additional, unknown pathway producing dsRNA. Hence, the possible interference of RNase3 with RNAi in the nucleus and the production of RNase3 via the postulated new pathway remains an interesting subject for further study.

Taken together, there is limited but increasing precedence for interaction between viral RNAi suppressors with SGS3 and its impact on RNAi. V2 of *Tomato yellow leaf curl virus* (family Geminiviridae) [20, 35], p2 of *Rice stripe virus* [21], TGBp1 of *Plantago asiatica mosaic virus* [18], protein P of *Lettuce necrotic yellows virus* [19], VPg of *Potato virus A* [11] and RNase3 of SPCSV represent examples reported from unrelated viruses. The unifying factor is that all six viral proteins are able to suppress only sense RNA–induced RNAi, indicating that they interfere with the steps of RNAi involving activities of RDR6 and SGS3. Hence, the results presented here advance understanding of the mechanisms needed for efficient RNase3-mediated suppression of RNAi and provide new information about the subcellular context and phase of the RNAi pathway in which RNase3 realizes RNAi suppression.

## Materials and Methods

### Cloning of the SGS3 coding sequence of *I*. *batatas*

An expressed sequence tag (SGN-U413895) from sweetpotato homologous to *sgs3* mRNA of tomato (*S*. *lycopersicum*; NCBI accession A5YVF1) and the tomato *sgs3* transcript sequences available in the SOL Genomics Network database (http://solgenomics.net/tools/blast/index.pl) were used as references during assembly of the *IbSGS3* coding sequence. The recently published high-throughput RNA-seq dataset of sweetpotato [26] was employed to retrieve the homologous sequences. An *in silico* translational analysis carried out on the assembled complete *IbSGS3* coding sequence revealed the expected conserved motifs and regions of homology common in the SGS3 proteins of different plant species [36]. Primers specific to *IbSGS3* were designed (S1 Table) according to the assembled sequence and used to amplify the coding region by PCR from sweetpotato cDNA, as described below.

*In vitro* plantlets of pathogen-free sweetpotato cv. Huachano were transferred to soil, grown in a growth chamber, and multiplied by taking stem cuttings. The growth conditions were: photoperiod 16 h, light intensity 200 μE m^−2^s^−1^; temperature 22–24°C (light) and 18–20°C (dark); relative humidity 40%. Plants were given fertilizer (N:P:K = 16:9:22) (Yara) mixed in water (0.3 g/l) in every watering. Leaves were harvested for RNA extraction when plants were 6 weeks old, frozen in liquid nitrogen, ground with a mortar and a pestle, and RNA extracted with Trizol (Ambion) as described [28]. RNA was dissolved in double-distilled water treated with DEPC and treated with DNase I to destroy DNA residuals according to the manual (Promega). Reverse transcription was carried out with *Moloney murine leukemia virus* reverse transcriptase (Promega) at 37°C for 1 h employing random hexamer primers. cDNA was subjected to PCR employing the *IbSGS3*-specific primers (IbSGS3XhoIfwd and IbSGS3XhoIrev; S1 Table) and using Phusion High Fidelity DNA polymerase (New England Biolabs). Amplified PCR products migrated at 1.3 kb, were excised from the agarose gel, purified using a PCR purification kit (Promega), and cloned in vector pCR-Blunt (Zero Blunt PCR cloning kit, Invitrogen). Several clones were sequenced. The *IbSGS3* coding sequence was deposited in GenBank (KJ364660).

The deduced amino acid sequence of IbSGS3 was aligned with the assembled putative SGS3 coding sequences of sweetpotato cv. Xushu 18 (SOL Genomics Transcriptome database) and other plant species using the MAFFT web server version 7 (http://mafft.cbrc.jp/alignment/server/). MAFFT produced a sequence alignment Clustal file that was modified in Microsoft Word. Phylogenetic relationships of different SGS3 proteins were analyzed and illustrated using MEGA5.05 software (S3 Fig).

### Protein expression and gene silencing

pCAMBIA1309 SPCSV RNase3-dsRED and pCAMBIA1309 SPCSV RNase3-Ala-dsRED binary vectors were used to express SPCSV RNase3 and SPCSV RNase3-Ala proteins translationally fused with dsRED at the C-terminus in *N*. *benthamiana* by agroinfiltration. RNase3 and RNase3-Ala coding sequences were amplified employing the pCAMBIA RNase3/Afwd and pCAMBIA RNase3/A (-stop) rev primers (S1 Table) from pET11d^+^ RNase3/RNase3Ala plasmids [3]. PCR products were purified, digested with *Sal*I and *Eco*RI, and cloned in pCAMBIA1390 mTalin-dsRED (provided by K. Boonrod, AlPlanta, Germany) linearized using the same restriction enzymes, which removed the m-Talin insert. pLH AtRDR6-mRFP was constructed for expression of an AtRDR6 that was translationally fused with mRFP for co-localization studies. *AtRDR6* was cloned in pLH-6K2-mRFP [27] and digested with *Xho*I, which removed the 6K2-encoding sequence. The vector for expressing AtRDR6-GFP (pBIC-AtRDR6-smGFP) was provided by Y. Watanabe, University of Tokyo, Japan. The Fib2-GFP construct was provided by M. Taliansky, James Hutton Institute, UK. pKB-HA-RDR6-GFP (provided by M. Wassenegger, AlPlanta, Germany) contained the *N*. *tabacum RDR6* sequence (NtRDR6) mutated at its active site and hence transcriptionally inactive. Expression was driven by *Cauliflower mosaic virus* 35S promoter and terminated by NOS terminator sequence. Expression of the construct resulted in translational fusion of hemagglutinin at the N-terminus and GFP at the C-terminus of NtRDR6.

Protein expression to test suppression of RNA silencing or analyze protein interactions by BiFC in leaves was carried out by agroinfiltration using *A*. *tumefaciens* C58C1:pGV3850. BiFC constructs were made based on the pLHYN and pLHYC plasmids [24] by cloning the gene to be studied in fusion with YN or YC to the *Nco*I site for a C-terminal tag and *Xho*I site for an N-terminal tag. The employed primers and produced constructs are in S2 Table. In the RNAi suppression assay, the *Agrobacterium* cultures were mixed for infiltration as follows: the cultures for expression of mGFP4 [25], RNase3 or RNase3-Ala, and IbSGS3-YN (BiFC-binary vector) or YN were diluted with infiltration medium to a final OD_600_ of 0.5 and combined at a 1:1:1 ratio. If any of these proteins was not used, the corresponding *Agrobacterium* strain was replaced with a strain expressing GUS [3]. GFP accumulation in the infiltrated leaves was observed under a hand-held UV-lamp (B-100 AP; UVP) daily up to 8 dpi. Images were taken with an EOS 40D digital camera (Canon) and processed using Corel PHOTO-PAINT X5 (Corel Corporation).

Total RNA was extracted from frozen leaf material with TRIzol (Invitrogen) according to the manufacturer’s instructions. Low and high molecular weight RNA fractions were separated as described [3]. Northern analysis for mRNA (3 μg of high molecular weight RNA) and siRNA (3 μg of low molecular weight RNA) were carried out using an antisense [a-32P]UTP-labeled (PerkinElmer) *gfp*-specific RNA probe as described [25]. Radioactive signals were detected using the IP screen (Kodak) and PhosphorImager (FLA-5001, Fuji).

### Protoplast preparation

Protoplasts were prepared from *N*. *benthamiana* wild-type infiltrated leaf tissue that had been transiently transformed with different expression constructs upon agroinfiltration. A half to full leaf of infiltrated leaf tissue was incubated with 5 ml enzyme solution (15 mg/ml cellulase, 2.5 mg/ml macerozyme, 7.5 mg/ml bovine serum albumin, 10% mannitol). The enzyme solution was stirred until completely clear, and then the pH was adjusted to 5.7 using HCl. The selected leaf pieces were cut into very thin stripes using a scalpel. The leaf material was immersed in the enzymatic solution in a 15-ml Falcon tube. Speed-vacuum filtration forced the leaf material to soak in the enzymatic solution. The tubes were placed in a light-proof box and incubated 2–3 h (depending on release of protoplasts) with gentle shaking. After incubation, the remaining leaf material was removed from the tube, and the solution containing protoplasts was subjected to centrifugation for 5 min at 400 × *g* with low speed-up/braking settings. Afterwards the enzymatic solution was removed, and wash buffer (154 mM sodium chloride, 125 mM calcium chloride, 5 mM potassium chloride, 4 mM 2-(N-morpholino)ethanesulfonic acid (MES), pH 5.7) was added such that the density of protoplasts was the same in each subsequent experiment; protoplast suspensions were used immediately.

### Epifluorescence and confocal microscopy

YFP fluorescence for BiFC was detected using a Zeiss AxioCam MRm epifluorescence microscope. Epidermal cells on the abaxial side of the agroinfiltrated leaves were viewed at 2 dpi using a YFP-compatible filter cube (GFP) and 40× objective. Images were captured and processed for presentation with Zeiss Zen lite 2012 program.

Confocal scanning microscopy was carried out at the Light Microscopy Unit, Institute of Biotechnology, University of Helsinki, using an SP2 AOBS confocal laser scanning system and a water immersion objective HCX PL APO ([63× OR 63x]/1.2 W) (Leica Microsystems GmbH) [37]. Leaf sections were cut from agroinfiltrated areas of leaves and mounted in water under a cover glass. Protoplasts were carefully pipetted into a chamber formed by a cover glass, objective glass, and tape. Consecutive optical sections were viewed to confirm overlap of fluorescent signals. For simultaneous detection of the different fluorophores and the chloroplast autofluorescence, specimens were scanned in between-lines mode using sequential settings. For GFP, chloroplasts, and dsRED, argon laser excitation was at 488 nm, and emission was monitored at 497–522 nm (GFP) and 690–740 nm (chloroplast autofluorescence); DPSS laser excitation was at 561 nm, with emission at 570–610 nm (dsRED). For detection of YFP and mRFP, the settings were as in [37]. All the settings were verified to show no crosstalk by scanning samples that expressed one fluorescent protein at a time. Experiments were repeated in at least three independent infiltrations for each assay.

### Analysis of tasiRNA

Total RNA was extracted from leaves of sweetpotato as described above and dissolved (0.5–1.0 mg/ml) in DEPC-treated water. High molecular weight and low molecular weight (LMW) RNA was isolated using a modified protocol for RNeasy Midi kit (Qiagen). After adding 4 volumes of RLT buffer (Qiagen), 4% β-mercaptoethanol, and 2.8 volumes of 99.5% ethanol, the RNA mix was loaded onto Midi columns (Qiagen) and centrifuged for 5 min at 4000 × *g* at room temperature. The eluted solution containing LMW RNA was transferred to a 15-ml tube and kept on ice. RPE buffer (2.5 ml) was added to the column, and after centrifugation for 2 min at 4000 × *g* at room temperature the eluted solution was collected and added to the same 15-ml tube. The LMW RNA fraction was precipitated by adding an equal volume of isopropanol. After incubation for 30 min on ice, the tubes were centrifuged at 12,000 × *g* at 4°C for 15 min. The pellet was washed with 75% ethanol and centrifuged for 5 min. RNA was resuspended in double-distilled water.

For tasiRNA detection, 50–70 μg LMW RNA extracted from sweetpotato cv. Huachano and the RNase3-transgenic lines RNase3JR1 and RNase3JR3 of this cultivar [2] was analyzed via 20% urea-PAGE (1× TBE buffer, 8 M urea, 20% polyacrylamide-bisacrylamide). Loading buffer contained 50% formamide and 50% 6× loading dye (Fermentas). RNA was heated at 95°C for 2 min, loaded onto the gel, and subjected to electrophoresis at 300 V. LMW RNA was transferred to a Hybond N^+^ membrane (Amersham) by semidry blotting (Bio-Rad) for 1 h at 20 V. The small RNAs were fixed on the membrane via incubation with a water-soluble carbodiimide crosslinker (Thermo Scientific).

A DNA probe (TGGGGTCTTACAAGGTCAAGA) was designed according to the highly conserved *TAS3* transcript expressed abundantly [*A*. *thaliana* TAS3 5´D7(+)] [28]. A probe (TGTGCTCACTCTCTTCTGTCA) detecting the abundantly expressed miRNA, miR156 [28], was used as a control. Probes were labeled using polynucleotide kinase (NEB) in a reaction containing 10× polynucleotide kinase buffer, 20 pmol oligos, 20 pmol [γ-^32^P]ATP (6000 Ci/mmol), 20 U polynucleotide kinase, and water to 20 μl. Each reaction was incubated for 30 min at 37°C. The probes were purified using Microspin columns (Bio-Rad). The membrane was prehybridized in hybridization buffer [5× saline sodium phosphate-EDTA, 5% SDS, 50% formamide, 5× Denhardt’s solution, 1 mg/ml herring sperm) at 35°C. For hybridization, the probe was heated at 95°C for 2 min and added to the hybridization buffer. Hybridization was carried out first with the TAS3 probe (overnight at 35°C). The blot was washed with buffer (2× saline sodium citrate, SSC; 0.1% SDS) twice for 30 min at 35°C. Signal was detected using the IP screen (Kodak) and PhosphorImager (FLA-5001, Fuji). Subsequently, the probe was stripped by washing the membrane several times in 0.1× SSC/1% SDS for 5 min at 90°C. Stripping was repeated until complete disappearance of radioactive signal (monitored after exposure to the IP screen for 1 h and analyzed with PhosphorImager). The membrane was then washed with 2× SSC/1% SDS and kept moist until hybridization solution with the miR156 probe was added and miR156 detection carried out as above. Signals were quantified using Bio-Rad Quantity One v4.6.9 software.

### Protein analysis

Agroinfiltrated leaf tissue was sampled 6 dpi, frozen in liquid nitrogen, and stored at –80°C. Leaf material (150–200 mg) was crushed in 400 μl of native extraction buffer (0.25 mM Tris-HCl, 0.005 mM EDTA, 1 mM PMSF, 0.1% ascorbic acid, 1% (w/v) Triton X-1000) and samples processed as described [27]. Samples were analyzed by 12% SDS-PAGE. PageRuler Plus Prestained Protein Ladder (Thermo Scientific) was used as a size marker. Immunodetection was carried out using monoclonal rabbit anti-GFP (dilution 1:5000) or polyclonal anti-RNase3 (1:500) as described [5].

## Supporting Information

S1 Fig Localization and co-localization of RNase3 and RDR6 in protoplasts derived from agroinfiltrated leaf tissue of *N*. *benthamiana* at 2 dpi. **(a)** RNase3 was expressed as a fusion with DsRed. **(b)** Co-localization of RNase3 (fused with DsRed) and AtRDR6 (fused with GFP). **(c)** Co-localization of RNase3 (fused with DsRed) with NtRDR6 (fused with GFP). TL, image with transmitted light. Scale bars, 10 μm.
(TIF)

S2 Fig Localization of the catalytically inactive mutant RNase3-Ala (fused with DsRed) expressed by agroinfiltration in epidermal cells of *N*. *benthamiana* and co-localization with AtRDR6 (fused with GFP). **(a)** Signals of RNase3-Ala were detected in the nucleus and cytoplasmic punctate bodies at the cell periphery. Chl, chloroplast autofluorescence (blue). **(b)** Signals for RNase3-Ala co-localized with AtRDR6 in cytoplasmic punctate bodies (illustrated at a different optical plane than in (a)). Scale bars, 10 μm.
(TIF)

S3 Fig Amino acid sequence comparison of SGS3 from different plant species. The coding sequence of IbSGS3 was amplified by PCR from cDNA of *I*. *batatas* cv. Huachano and verified by sequencing of several clones. The SGS3 sequences of other plant species were obtained from www.uniprot.org. **(a)** CLUSTAL alignment of amino acid sequences using MAFFT (v7.023b). The part of sequences framed with a dashed line includes the XS domain that contains amino acid residues characteristic of SGS3 (NCBI, RRM-like XS domain in plants, cd12266). **(b)** Phylogenetic analysis of SGS3 sequences carried out with the Neighbor Joining algorithm in MEGA5.05. AtSGS3: *Arabidopsis thaliana* SGS3 (UniProt Q9LDX1); SlSGS3: *Solanum lycopersicum* SGS3 (UniProt A5YVF1); NtSGS3,a and NtSGS3,b: two SGS3 homologs of *Nicotiana tabacum* (UniProt L8B8E8 and L8B897, respectively); IbSGS3: *Ipomoea batatas* SGS3 (cloned and sequenced from cv. Huachano in this study); ZmSGS3: *Zea mays* SGS3 (UniProt A1Y2B7); OsSGS3: *Oryza sativa* SGS3 cv. Indica (UniProt A2ZIW7) and cv. Japonica (UniProt Q2QWE9). Only bootstrap values higher than 90% (of 100 replicates) are shown. Scale indicates Kimura units (Tamura *et al*., 2007; *Mol Biol Evol* **24**, 1596–1599).
(TIF)

S4 Fig Analysis of *TAS3* siRNA in sweetpotato cv. Huachano and two RNase3-transgenic lines of the cultivar. (a) Detection of 21-nt tasiRNA and miRNA using probes for the conserved sequence of the most abundant tasiRNA (*A*. *thaliana* 5´D7(+); position 7 from miR390 cleavage site) and miRNA156, respectively. Lanes 1 and 2, sweetpotato cv. Huachano; lanes 3 and 4, two plants of the RNase3-transgenic line RNase3jR1 of cv. Huachano; lane 5, RNase3-transgenic line RNase3jR3 of cv. Huachano. The transgenic lines expressed RNase3 under the enhanced 35S promoter (denoted as j) (Cuellar *et al*., 2009; *Proc Natl Acad Sci USA* **106**, 10354–10358). Detection of miR156 served as an internal control and loading marker. (b) Quantification of radioactive signals using Bio-Rad Quantity One v4.6.9 software. Signals obtained by hybridization with *A*. *thaliana* TAS3 5´D7(+) probe were normalized to signals of the respective control (signals obtained with AtmiR156 probe). The total pixel value was set to an arbitrary unit of 100. WT, wild-type cv. Huachano; R3R1, transgenic line RNase3jR1; R3R3, transgenic line RNase3jR3.
(TIF)

S1 Table Primers used in PCR. (DOCX)

S2 Table Plasmids made for bimolecular fluorescence complementation assays (all constructs were included in experiments). Restriction sites are underlined.
(DOCX)
