# Supplementary figures and images for: Viral RNase3 Co-Localizes and Interacts with the Antiviral Defense Protein SGS3 in Plant Cells
Source: PLoS One. 2016 Jul 8;11(7):e0159080. doi: 10.1371/journal.pone.0159080 (PMC4938523; doi:10.1371/journal.pone.0159080)

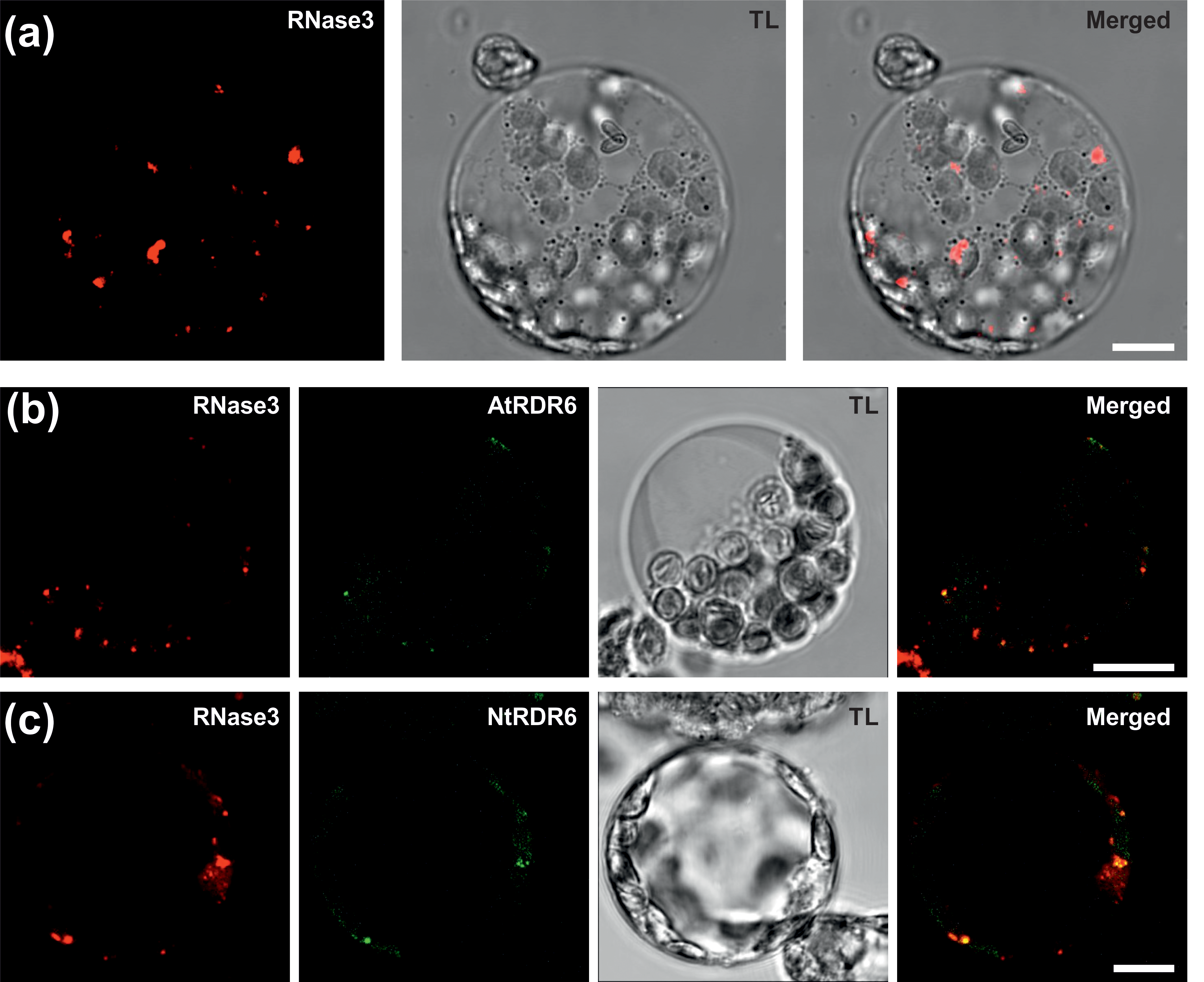

Supplement: S1 Fig — (a) RNase3 was expressed as a fusion with DsRed. (b) Co-localization of RNase3 (fused with DsRed) and AtRDR6 (fused with GFP). (c) Co-localization of RNase3 (fused with DsRed) with NtRDR6 (fused with GFP). TL, image with transmitted light. Scale bars, 10 μm. (TIF) [file pone.0159080.s001.tif]

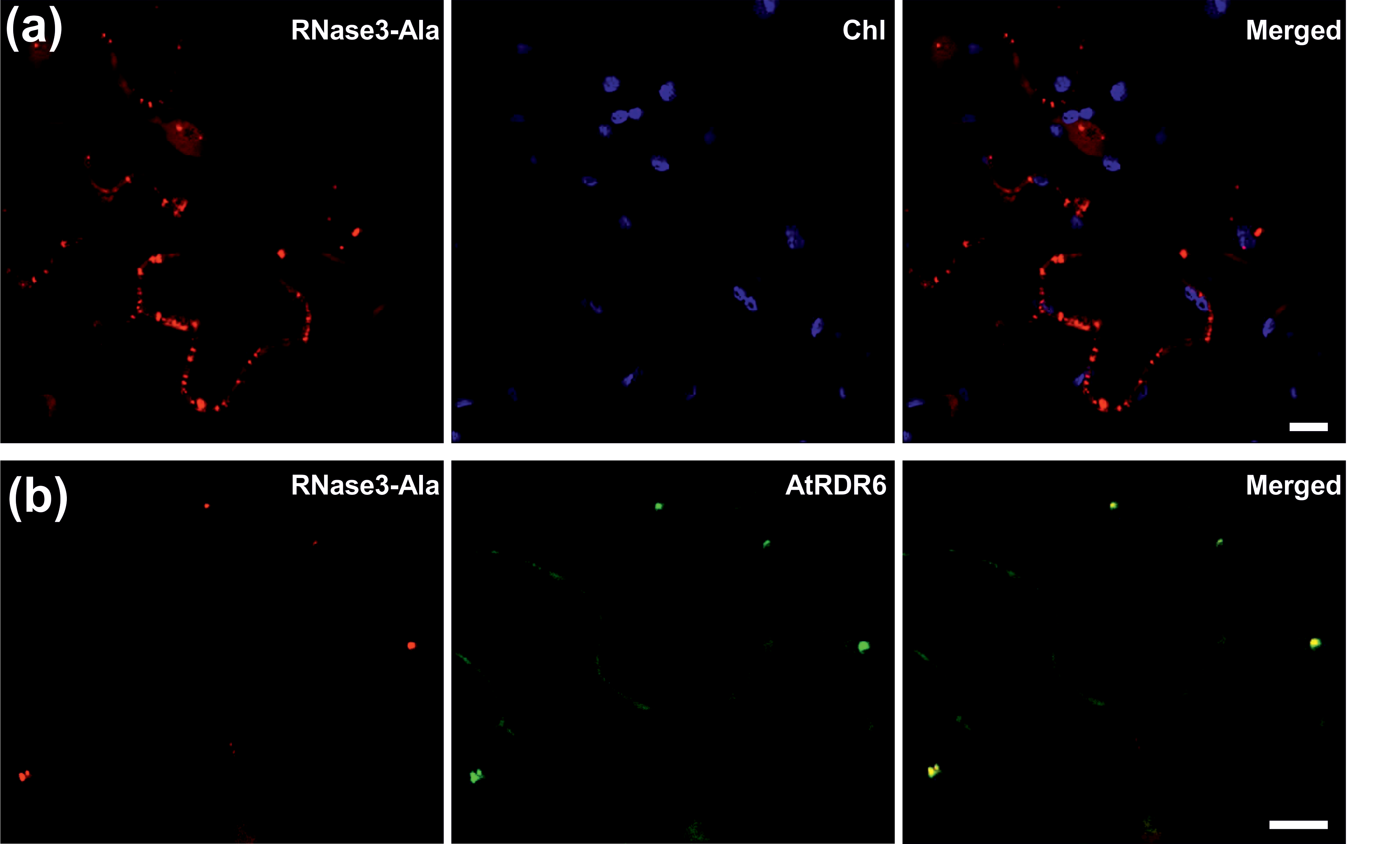

Supplement: S2 Fig — (a) Signals of RNase3-Ala were detected in the nucleus and cytoplasmic punctate bodies at the cell periphery. Chl, chloroplast autofluorescence (blue). (b) Signals for RNase3-Ala co-localized with AtRDR6 in cytoplasmic punctate bodies (illustrated at a different optical plane than in (a)). Scale bars, 10 μm. (TIF) [file pone.0159080.s002.tif]

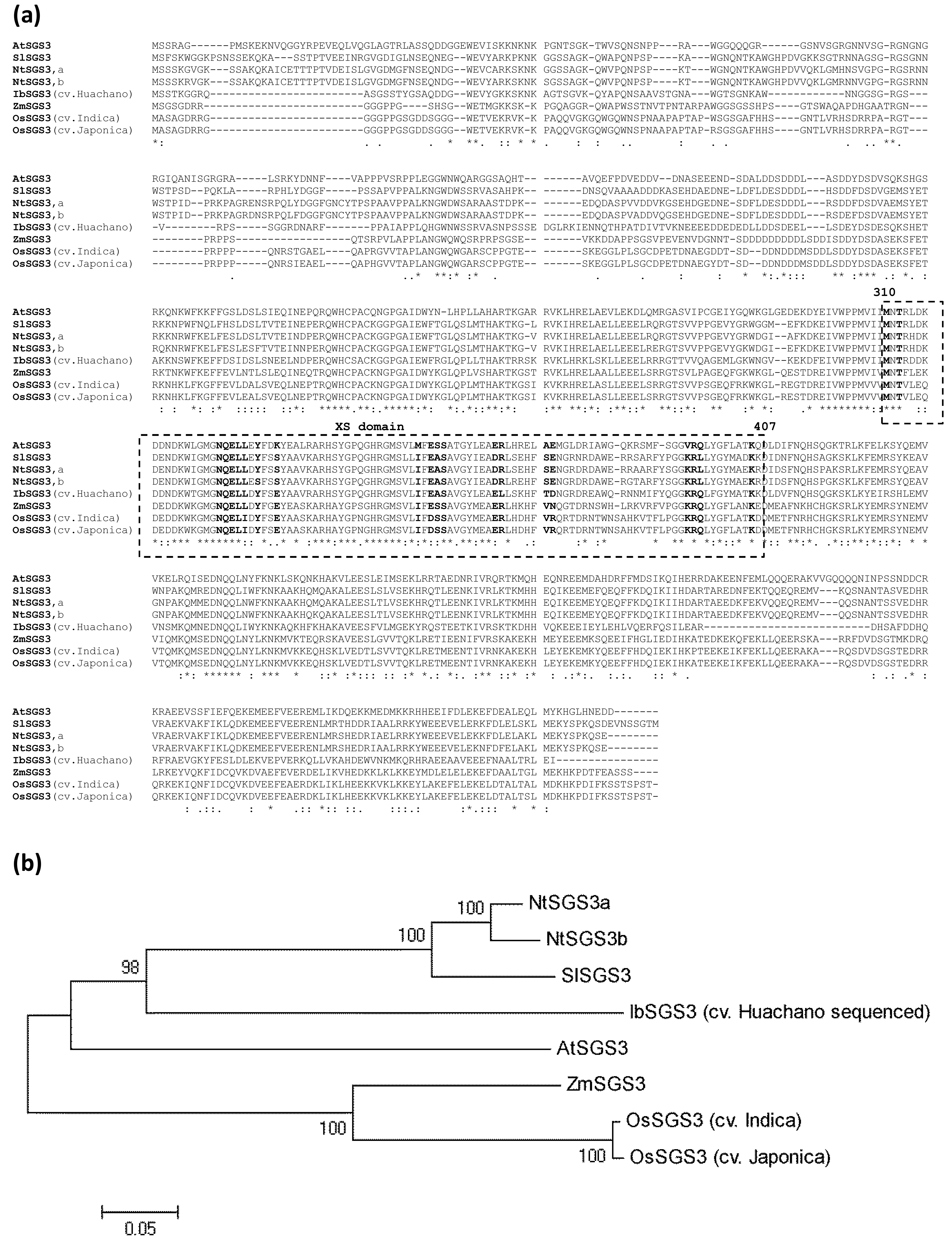

Supplement: S3 Fig — The coding sequence of IbSGS3 was amplified by PCR from cDNA of I. batatas cv. Huachano and verified by sequencing of several clones. The SGS3 sequences of other plant species were obtained from www.uniprot.org. (a) CLUSTAL alignment of amino acid sequences using MAFFT (v7.023b). The part of sequences framed with a dashed line includes the XS domain that contains amino acid residues characteristic of SGS3 (NCBI, RRM-like XS domain in plants, cd12266). (b) Phylogenetic analysis of SGS3 sequences carried out with the Neighbor Joining algorithm in MEGA5.05. AtSGS3: Arabidopsis thaliana SGS3 (UniProt Q9LDX1); SlSGS3: Solanum lycopersicum SGS3 (UniProt A5YVF1); NtSGS3,a and NtSGS3,b: two SGS3 homologs of Nicotiana tabacum (UniProt L8B8E8 and L8B897, respectively); IbSGS3: Ipomoea batatas SGS3 (cloned and sequenced from cv. Huachano in this study); ZmSGS3: Zea mays SGS3 (UniProt A1Y2B7); OsSGS3: Oryza sativa SGS3 cv. Indica (UniProt A2ZIW7) and cv. Japonica (UniProt Q2QWE9). Only bootstrap values higher than 90% (of 100 replicates) are shown. Scale indicates Kimura units (Tamura et al., 2007; Mol Biol Evol 24, 1596–1599). (TIF) [file pone.0159080.s003.tif]

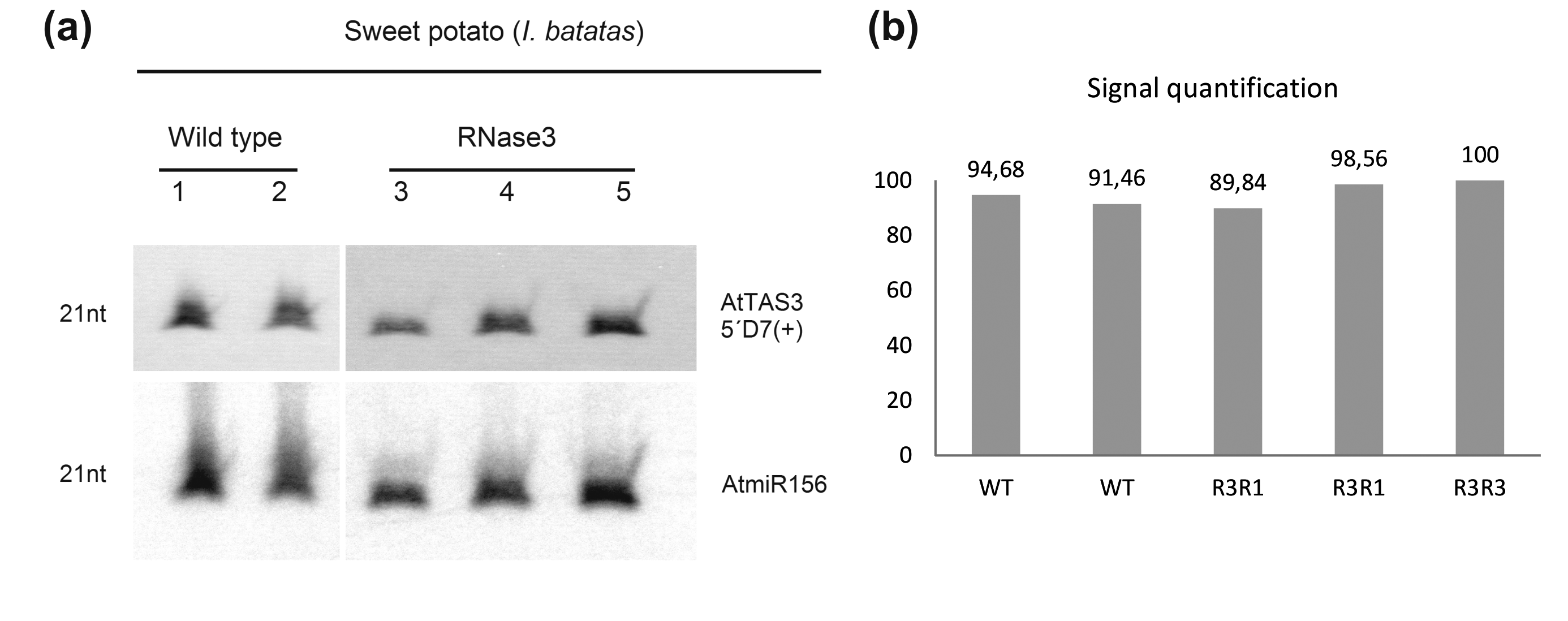

Supplement: S4 Fig — (a) Detection of 21-nt tasiRNA and miRNA using probes for the conserved sequence of the most abundant tasiRNA (A. thaliana 5´D7(+); position 7 from miR390 cleavage site) and miRNA156, respectively. Lanes 1 and 2, sweetpotato cv. Huachano; lanes 3 and 4, two plants of the RNase3-transgenic line RNase3jR1 of cv. Huachano; lane 5, RNase3-transgenic line RNase3jR3 of cv. Huachano. The transgenic lines expressed RNase3 under the enhanced 35S promoter (denoted as j) (Cuellar et al., 2009; Proc Natl Acad Sci USA 106, 10354–10358). Detection of miR156 served as an internal control and loading marker. (b) Quantification of radioactive signals using Bio-Rad Quantity One v4.6.9 software. Signals obtained by hybridization with A. thaliana TAS3 5´D7(+) probe were normalized to signals of the respective control (signals obtained with AtmiR156 probe). The total pixel value was set to an arbitrary unit of 100. WT, wild-type cv. Huachano; R3R1, transgenic line RNase3jR1; R3R3, transgenic line RNase3jR3. (TIF) [file pone.0159080.s004.tif]
