# Supplementary material for: Viral RNase3 Co-Localizes and Interacts with the Antiviral Defense Protein SGS3 in Plant Cells
Source: PLoS One. 2016 Jul 8;11(7):e0159080. doi: 10.1371/journal.pone.0159080 (PMC4938523; doi:10.1371/journal.pone.0159080)
Supplement: S1 Table — (DOCX) [file pone.0159080.s005.docx]

**S1 Table.** Primers used in PCR.

| **Primer** | **Sequence (5’-3’)** | **Template** |  |
| --- | --- | --- | --- |
| pCAMBIA RNase3/Afwd | CTATTTACAATTACAGTCGACAT | pET 11d^+^ SPCSV RNase3/-Ala | |
| pCAMBIA RNase3/A (-stop) rev | CTCGGAGGAGGCCATGAATTCACTCAGATTTAGAGCTTCA | pET 11d^+^ SPCSV RNase3/-Ala | |
| IbSGS3-XhoIfwd* | CCGCTCGAGATGAGTTCGACCAAAGGGGT | Sweetpotato cDNA | |
| IbSGS3-XhoIrev* | CCGCTCGAGAATCTCCAACCTAGTTAATG | Sweetpotato cDNA | |

*The PCR program included initial denaturation at 98°C for 1 min and 36 cycles of denaturation at 98°C for 10 s, primer annealing at 54°C for 23 s, and elongation at 72°C for 40 s, with final extension at 72°C for 5 min.
