# Supplementary material for: Viral RNase3 Co-Localizes and Interacts with the Antiviral Defense Protein SGS3 in Plant Cells
Source: PLoS One. 2016 Jul 8;11(7):e0159080. doi: 10.1371/journal.pone.0159080 (PMC4938523; doi:10.1371/journal.pone.0159080)
Supplement: S2 Table — Restriction sites are underlined. (DOCX) [file pone.0159080.s006.docx]

**S2 Table.** Plasmids made for bimolecular fluorescence complementation assays (all constructs were included in experiments). Restriction sites are underlined.

| **Plasmids** | **Primer sequences (5’–3’)** | **Template** |
| --- | --- | --- |
| YN-SPCSV RNase3(Ala)/  YC-SPCSV RNase3(Ala) | R3-NcoIF: ttaccatgggcaatgatcctgatggttccgatttattc  R3-NcoIR: ttaccatggctaactcagatttagagcttcaacag | pET 11d^+^ SPCSV RNase3 |
| SPCSV RNase3-YN/  SPCSV RNase3-YC | R3-XhoIF: aatctcgagatgatcctgatggttccgatttattc  R3-XhoIR: attctcgagtgcactcagatttagagcttcaacag | pET 11d^+^ SPCSV RNase3 |
| AtSGS3-YN/AtSGS3-YC | SGS3-XhoIF: aatctcgagatgagttctagggctggtc  SGS3-XhoIR: attctcgagtgcatcatcttcattgtgaagg | LIC/pYL AtSGS3 (ABRC)* |
| IbSGS3-YN/IbSGS3-YC | IbSGS3-NcoIF: atgccatggcaatgagttcgaccaaaggggt  IbSGS3-NcoIR:catgccatggtctctccaacctagtta  IbSGS3XhoIF: ccgctcgagatgagttcgaccaaaggggt  IbSGS3XhoIR:ccgctcgagaatctccaacctagttaatg | pCR blunt IbSGS3 |
| AtRDR6-YN/AtRDR6-YC | RDR6-XhoIF: ttactcgagatggggtcagagggaaatatg  RDR6-XhoIR: ttactcgagtgcgagacgctgagcaagaaact | pBIC-AtRDR6-smGFP (provided by Y. Watanabe) |
|  |  |  |

**A. thaliana* SGS3 was obtained from Arabidopsis Biological Resource Center (ABRC, http://www.arabidopsis.org, Stock number DKLAT5G23570.1, Dinesh-Kumar and Snyder).
